# Importance of suberin biopolymer in plant function, contributions to soil organic carbon and in the production of bio-derived energy and materials

**DOI:** 10.1186/s13068-021-01892-3

**Published:** 2021-03-20

**Authors:** Anne E. Harman-Ware, Samuel Sparks, Bennett Addison, Udaya C. Kalluri

**Affiliations:** 1 Renewable Resources and Enabling Sciences Center, Center for Bioenergy Innovation, National Renewable Energy Laboratory, Golden, CO 80401 USA; 2 Biosciences Division and Center for Bioenergy Innovation, Oak Ridge National Laboratory, Oak Ridge, TN 37830 USA

**Keywords:** Suberin, Biopolymer, Biomaterial, Biomass, Cork, Root, Soil, Carbon, Genomics, Bioenergy

## Abstract

Suberin is a hydrophobic biopolymer of significance in the production of biomass-derived materials and in biogeochemical cycling in terrestrial ecosystems. Here, we describe suberin structure and biosynthesis, and its importance in biological (i.e., plant bark and roots), ecological (soil organic carbon) and economic (biomass conversion to bioproducts) contexts. Furthermore, we highlight the genomics and analytical approaches currently available and explore opportunities for future technologies to study suberin in quantitative and/or high-throughput platforms in bioenergy crops. A greater understanding of suberin structure and production in lignocellulosic biomass can be leveraged to improve representation in life cycle analysis and techno-economic analysis models and enable performance improvements in plant biosystems as well as informed crop system management to achieve economic and environmental co-benefits.

## Background

### Suberin production and function in plants

The survival of terrestrial plants depends on their ability to control water loss and solute transport, insulate from climatic extremes and variations, protect against pathogenic attacks and to recover from mechanical damage. Suberin is a lipophilic bio-macromolecule that is integral for the ability of plants to withstand and recover from such stresses and challenges [1–7]. Suberin biosynthesis, regulation and associated plasticity under various conditions determine qualitative and quantitative properties of suberin and its influence on plant physiological and structural properties in both above- and below-ground tissues. Suberin abundance varies according to plant and tissue types, developmental stage and plant’s ability to respond to environmental changes. For example, suberin content of *Quercus suber* (cork) bark is about 30–50% of the dry weight mass [8, 9] whereas in skins of carrot, beets and potato, suberin content can range from 20–50% [10]. Holloway compared various lignocellulosic biomass types and found suberin content to range widely depending on biomass type from as low as 8% to as high as 60% extracted dry weight % of material [11]. Given that the biological purpose of suberin occurrence and production is to provide a protective barrier in plant cell walls, cell wall-derived suberin is among the persistent plant components found in soil [12–14]. Suberin derived from plant-detritus is therefore of interest as a biogeochemical biomarker to estimate the potential contributions from plants to soil organic matter (SOM) and recalcitrant carbon, C, with longer residence times in soil [15–18].

The production of suberin can change in response to drought and other abiotic stresses to prevent water loss and enhance water retention capacity within root systems [7]. Suberin can also impact plant mineral absorption and transport, and therefore, nutrition, by acting as a solute diffusion barrier. Generally, root suberization, increased or ectopic, has been linked to a decrease in the accumulation of Ca, Zn and Mn, and an increase of Na, S, K, Mo, As and Se [19–22]. Suberin has also been linked to aiding plant resistance to biotic stresses, such as microbial pathogen attack [6]. For example, the phenolic and aliphatic domains of suberin in potato periderm wound response were found to be important in disease resistance to specific bacterial and fungal pathogens, respectively [23]. The aliphatic component of root suberin also negatively correlated with soybean plant mortality to a fungal pathogen, *Phytophthora sojae* [24]. Suberin production is also impacted in response to environmental stresses [14, 25] and its accumulation has been linked to prevention of radial oxygen loss in root systems [26]. Changes in environmental conditions, specifically elevated carbon dioxide (CO_2_) and temperature, have been reported to result in altered suberin chemistry of roots [15]. A recent elegant study provided evidence for reciprocal effects of root endodermal diffusion barrier (suberin) and microbiota, showing how root microbiota impact suberin deposition, and plant ionome; and that the functional role of suberin as diffusion barrier in turn determines microbiome composition [27]. Suberin production and deposition in roots have clear implications on plant physiology, growth, interactions with microbes and stress adaption; and have potential ecosystem level impacts in the contexts of root organic matter turnover, soil chemical composition, moisture, and other factors relevant to microbiome dynamics.

Suberized cells can be found in the stem periderm and the root periderm, exodermis and endodermis, and other specialized tissues such as seed coat, fruit and vegetable skin, and abscission zone [22, 28–30]. Suberin lamellae in root endodermis are typically deposited as secondary walls after the development of Casparian bands [31]. The major biopolymer component of lamellae is suberin with lignin occasionally reported as a minor component in monocots [32]. Suberin lamellae have been characterized as having alternating electron-lucent and electron-dense layers, which consist of a suberin polyaliphatic domain and a suberin polyphenolic domain [33].

Genetic understanding of suberin biosynthesis in stem and root and in other specialized tissues such as seed coats, and their functional roles in water movement regulation, defense and mineral accumulation properties has been greatly aided by *Arabidopsis* mutant characterization studies [7, 22, 28, 29]. While transport proteins constitute a well-known, central mechanism by which roots regulate uptake of materials (nutrients, solutes, etc.), there is also a level of regulation on material uptake in the non-specific apoplastic transport pathway between cells. Suberin-rich “barriers” are central to the regulation of apoplastic transport with root endodermal suberin inhibiting apoplastic movement of both water and solutes into the stele, and a similar role for exodermal suberin at root surface [22, 29, 31].

Altered levels of suberin content can result in significant impacts on plant health and productivity [22]. A detailed characterization of the *Arabidopsis enhanced suberin1(esb1)* mutant provided clear evidence not only for root suberin in water and solute transport, but also that higher root suberin content had ramifications on whole plant function including a reduction in water loss and wilting under drought-like conditions, and differential shoot ionome composition [22]. Given that quantitative differences in suberin levels also have effects on plant functions, such as variation in permeability of the apoplast to both water and solutes, changes in suberin quality and/or quantity can in turn impact growth and composition of plant shoot.

Considering drought, a die-back of cortical and epidermal tissues and increased suberization of endodermis protecting the stele from desiccation has been reported from *Lolium* plants  [34]. In response to high salinity, root systems can reduce their growth rate while enhanced endodermal and exodermal suberization can occur closer to the root apex [31, 35]. Waterlogging typically results in soil oxygen depletion, changes in soil microbial activity, increased pathogenic microbe and toxic microbial bi-products in the soil media (harmful organic acids, lowered redox potentials or phytotoxic compounds) [6, 36, 37]. As another adaptive advantage in waterlogged conditions, the suberized apoplastic barrier minimizes radial oxygen loss, enhancing the diffusion of oxygen towards the root apex and impeding penetration of toxins or pathogens into the roots [31, 38, 39].

Toxins, nutrient status and CO_2_ levels in the environment can influence tissue development as well as cell wall chemistry, including suberization [15, 31]. For example, corn seedlings grown under magnesium (Mg)-deficient conditions were found to be more suberized in the endo- and hypodermis/exodermis relative to control conditions [40]. Suberin as the hydrophobic component of the apoplastic barrier plays a critical role in plant damage under abiotic (e.g., waterlogging) and biotic (e.g., pathogen attack) stresses [41]. The extent to which timing, location and abundance of suberin deposition and suberin composition contributes to the formation and properties of the apoplastic barrier, across various plant types, is an ongoing area of research.

### Suberin structural and compositional analyses

Suberin is a nonlinear, irregular, poly(acylglycerol) macromolecule built from poly-functional long chain fatty acids, fatty alcohols and glycerol which are covalently linked to phenolic moieties. A general structure of suberin was proposed by Kolattukudy in which a cross-linked aromatic subdomain is covalently linked to long-chain diacids and hydroxyacids through ester bonds [41, 42]. *ω*-hydroxyacids and *α*,*ω*-diacids are typically the most abundant long chain lipids found in suberin [1, 8]. It was later considered that in addition to phenolics and long-chain fatty acids, glycerol is an additional suberin monomeric unit [43–45]. In strong support of the hypothesis that glycerol units act to cross-link a ferulate-rich polyaromatic domains with long-chain hydroxyacids, Correia et al. identified monoacylglycerol, diacylglycerol and triacylglycerol units and further elucidated the specific lipid and phenolic moieties within native and near-native isolated cork suber using a suite of solution-state nuclear magnetic resonance spectroscopy (NMR) data in conjunction with microscopic methods and mass spectrometry [46].

Suberin (primary association with cork) and cutin (primary association with cuticle) are both complex macromolecules that serve as protective barriers in plants. While suberin is a biopolymer consisting of both aromatic and aliphatic domains, cutin is a polyester consisting primarily of omega hydroxy acids (C16 and C18 families) and has lower abundance of longer chain fatty acids (C20–C30) than suberin [42]. In contrast to polyaromatic lignin, suberin is characterized by the presence of higher levels of hydroxycinnamic acids and derivatives (e.g., ferulates).

Due in part to its heterogeneous, irregular and diverse nature, unaltered suberin isolation from plant tissues and characterization remains an analytical challenge in research applications. Additionally, the nature and properties of suberin and derived moieties make detailed characterization tedious and laborious, often requiring many steps, chromatographic separation and sophisticated detection technology, rendering high-throughput and accurate analyses difficult to achieve. Typically, acid or base-catalyzed transesterification or methanolysis are used to remove or isolate and determine suberin content and its lipid, phenolic and glycerol components in biomass but many of these methods may induce changes on the structure and may not accurately reflect relative composition of specific constituents and moieties [8–10]. The structure and composition of altered and native suberin and its components in biomass tissues have been studied using a variety of microscopy techniques, mass spectrometry (MS), NMR and other spectroscopic characterization methods. Table 1 summarizes several methodological approaches used to characterize suberin content in biomass as well as its structure and monomeric constituents.

**Table 1:** Summary of analytical methodologies used to analyze suberin structure and composition

Technique	Methods/experiments/types/parameters	Results or findings	References
Wet Chemistry	Acid/base-catalyzed transesterification/methanolysis	Total suberin content, depolymerized components	[8–10, 53]
Microscopy	TEM	Lamellar structure of cell wall	[47]
	SEM	Isolated suberin macromolecular structure	[46]
	Confocal, fluorescence, histological staining	Suberin presence and relative abundance in cell walls and tissues	[48–52]
NMR	Solution-state NMR, solid-state NMR	Aliphatics, phenolics and glycerol are components of suberin	[43, 45, 69, 73, 166–168]
	Solution-state NMR, solid-state NMR	Polyaromatic and phenolic composition, hydroxycinnamates including ferulic acid, lignin-like subunits	[75, 102, 169–173]
	Solid-state NMR relaxation and dynamics studies	Two distinct methylene CH_2_ environments identified with differing dynamics, aliphatic acylglycerols with mid-chain modified hydrocarbons found in more rigid core, saturated alkanols and alkanoic acids are more dynamic and spatially distinct	[69–73, 79, 174]
	Solution-state NMR, gel-state NMR	Primary structure: how monomeric units are linked to polymeric / oligomeric units	[46, 74, 160]
	Solid-state NMR	Overall superstructure and domain architecture	[70, 72, 79, 160]
Mass Spectrometry	GC/MS of depolymerized components	Phenolics, lipids, glycerol components as monomers	[9, 53, 57]
	LC/MS of depolymerized components	Phenolics, lipids, glycerol components as monomers and oligomers	[54]
	Pyrolysis-mass spectrometry of biomass	Fingerprinting for phenolics and lipid components, screening for thermochemical conversion paradigms	[59, 150]
	Pyrolysis-mass spectrometry of soils	Fingerprinting for phenolics and lipid components, biomarker for plant species in soils	[60–68]
	MALDI-MS	Suberin structure in tissue and separate hydrolysate analysis for phenolics, lipids	[56]

A working hypothesis of suberin superstructure and high-level domain architecture as it exists in plant cells arises from a number of dominant spectroscopic and microscopic observations. First, transmission electron microscopy (TEM) images of suberized plant cell walls reveal a poly-lamellar structure with repeating dark and light bands, with 30–60 repeating layers found in cork [47]. Isolated suberin precipitated in water subsequently analyzed by TEM and scanning electron microscopy (SEM) exhibited elongated polygon structures with preserved glycerol backbones and overall lengths of 100–175 μm [46]. Also, staining methods have been used to indicate the presence of both aliphatic and aromatic components and domains in suberin [41, 42]. Lulai and Morgan described potato tuber wound suberin as being deposited in separate hydrophobic/lipid and phenolic/lignin processes based on microscopy analysis in conjunction with neutral red and berberine cytochemical probes [48]. The authors noted that the neutral red was specific for the hydrophobic/lipid domain of suberin and, based on results used in conjunction with berberine, they suggested the deposition of lipid and phenolic domains in suberin occured in separate processes [48]. Lux et al. described a clearing and staining methodology using free-hand sections and whole-mount samples for observation of suberin in endodermal root cells of *Arabidopsis* [49]. The authors describe the optimization of the staining procedures for various tissues in different plant types and describe suberin observations based on berberine and fluorol yellow for lamellar suberin specificity and also include post-staining procedures using aniline blue, toluidine blue O as well as safranin O to better visualize exodermal, epidermal and endodermal cells [49]. Classic histological staining methods, particularly including dyes Nile red (specific for suberin) and auramine O (binds various substrates including suberin, cutin and lignin), have also been used with “ClearSee” clearing protocols to analyze suberin in *Arabidopsis* roots using confocal microscopy [50]. Cohen et al. used histochemistry, fluorescent protein tags and confocal laser scanning microscopy to demonstrate SUBERMAN transcription factor regulation in endodermal cells with varying degrees of suberization as well as TEM to show suberin lamellae in epidermis, cortex and endodermis in *Arabidopsis* roots [51]. AtMYB107 transcription factor was also shown to regulate suberin biosynthesis in *Arabidopsis* seed coats, but not root suberin biosynthesis or cutin biosynthesis, based on supporting data from histochemical staining (Sudan black B for lipids including waxes hydrophobic domains in suberin as well as toluidine blue for analysis of leaf cuticles) in conjunction with confocal microscopy, SEM and TEM [52].

Specific suberin monomers or constituents, principally fatty (saturated, unsaturated and substituted) acids, fatty (or aliphatic) alcohols, mono- and di-*ω*-hydroxyacids, *α*,*ω*-diacids, epoxy-substituted lipids, phenolics and glycerol have been characterized by various mass spectrometry techniques, particularly on depolymerized or otherwise isolated or extracted suberin material (Tables 1 and 2). A summary of suberin monomeric components and structures is provided in [1]. Extensive tables of MS data, particularly from GC/MS, specifying and often quantifying specific suberin constituents can be found in the literature (for example Corriera et al. [46], Holloway [11]). Here, we have provided a brief outline in Table 2 of broad categories of various suberin moieties and several types of biomass and tissues where their occurrence has been identified with corresponding literature and methods used to identify the species. Generally, suberin lipids (fatty acids, alcohols and diacids with various functional groups such as additional hydroxyl or epoxy groups) have been identified in fruit cuticles, tree barks, and in roots and leaves of many plant types. The abundance and distribution of suberin-derived lipids of chain lengths C7–C32 varies in abundance depending on plant type and tissues where the most abundant species are typically C16–C26 chain lengths. Phenolic-derived species typically consist of ferulic and benzoic acids which also vary depending on plant and tissue type. Glycerol recovered from suberin analysis also varies in abundance but is typically a large portion (on the order of 20 mass % suberin) of the suberin content [53].

**Table 2:** Summary of major suberin moieties found in particular plant species and tissues and associated references

Plant species	Tissues	Suberin moieties	Method, references
Tomato, nectarine, apple	Cuticle	C16–C22 fatty acids, *α*,*ω*-diacids, *ω*-hydroxy acids, aliphatic alcohols, epoxy-substituted acids	MALDI-MS [56]
Potato	Peel Wastes	C12–C30 fatty acids, *α*,*ω*-diacids, *ω*-hydroxy acids, aliphatic alcohols, hydroxycinnamic acids	Py-GC/MS [150]
*Quercus suber*	Cork (bark)	C22–C28 fatty alcohols, C14–C26 fatty acids, C16–C26, *ω*-mono and di-hydroxy acids, C7–C26 *α*,*ω*-diacids, epoxy acids, phenolics (primarily ferulic, benzoic, coumaric and vanillic acids), glycerol, tri- and di-glyceride structures	GC/MS, NMR [9, 46, 53, 59]
Birch	Bark	C16–C22 hydroxylated fatty acids, *α*,*ω*-diacids, ferulic acid	GC/MS, NMR [143]
*Quercus robur, Q. ilex, Q. suber, Fagus sylvatica, Castanea sarica, Betula pendula, Acer griseum, Fraxinus excelsior, Acer pseudoplatanus, Ribes nigrum, Euonymus alatus, Populus tremula, Solanum tuberosum, Sambucus nigra, Laburnum anagyroides, Cupressus leylandii*	Cork, bark	Aliphatic alcohols, C16–C32 fatty acids, *ω*-mono and di-hydroxy acids (C16–C26), *α*,*ω*-diacids, epoxy-substituted acids (C18)	GC/MS [11]
Root vegetables (beet, parsnip, carrot, sweet potato, rutabaga, turnip)	Skin	C14–C32 fatty acids, C15–C24 *α*,*ω*-diacids, C16–C28 *ω*-hydroxy acids, C18–C30 aliphatic alcohols	GC/MS [10]
Soil		*α*,*ω*-acids, *ω*-hydroxy acids, C16–C34 fatty acids, aliphatic alcohols, coumaric and ferulic acids	Py-GC/MS [12, 64], Py-FIMS [60], GC/MS [65]
Sycamore, spruce, cork	Bark	C14–C26 fatty acids, *α*,*ω*-diacids, hydroxy acids, aliphatic alcohols, ferulic acid, benzoic acid	GC/MS [138]

GC/MS analyses require that the suberin be depolymerized and monomeric constituents are derivatized prior to analysis. Additionally, liquid chromatography with mass spectrometry (LC/MS) may be used to identify and quantify suberin-derived products. Thiombiano et al. developed a workflow to characterize flax seed coat hydrolysates, including suberin and cutin-derived species as well as lignans, using LC/MS and GC/MS [54]. Their methodology accounted for the production and analysis of partial hydrolysates to analyze oligomers in an attempt to sequence the macromolecular network of the various biopolymers.

Other techniques such as matrix assisted laser desorption ionization mass spectrometry (MALDI-MS) are important for analyzing biopolymers such as suberin given their ability to probe structural information. For example, MALDI-MS was used to analyze fruit cuticles to image the surface heterogeneity and to understand the structural features associated with cutin and suberin in tissues [55, 56]. The authors used in situ hydrolysis of the suberin and cutin to obtain spectral characteristics of the isolated biopolymer hydrolysates and the de-suberized tissues.

While GC/MS, LC/MS and MALDI-MS are powerful and important techniques used to analyze biopolymers such as suberin, they often require laborious sample preparation and chemical depolymerization steps that are not easily adaptable to high-throughput analyses that may be needed for population-scale and multi-omic studies. Mass spectrometry techniques such as pyrolysis-mass spectrometry (py-MS) offer the advantage of potential minimal sample preparation, although these techniques often require time intensive chromatographic separation for speciation and quantitative, unbiased characterization. Additionally, py-MS techniques may not necessarily provide structural insights but could potentially be implemented in a high-throughput platform. Py-MS techniques use a pyrolysis step (thermal decomposition in the absence of oxygen) to produce vapors from materials prior to chromatography and/or MS analysis. Marques et al. demonstrated how py-GC/MS can be used to analyze suberin in biomass and the complications that can arise related to the presence of lignin and the analytical conditions and parameters used [57]. Py-GC/MS has been used to analyze potato peels and their fermented wastes to simultaneously characterize the materials and inform thermochemical process utilization potential based on the products generated [58]. Pyrolysis with a methylating agent followed by GC/MS analysis was used to study *Quercus suber* cork and its isolated suberin as well as lignin fractions in conjunction with NMR experiments [59]. The authors suggest that ferulates may act as a cross-linking unit between lignin and suberin carbohydrates in cork cell walls.

Py-MS techniques such as pyrolysis field ionization mass spectrometry (Py-FIMS) are particularly useful for analyzing soils and have been used to characterize soils based on the suberin species detected in the analyses [12, 13, 60–63]. As with biomass, soils may also be treated with methylating agents prior to py-MS to aid in the production of volatile vapors. Nierop reported the py-GC/MS analysis of soils with thermally assisted hydrolysis and methylation to characterize suberin and cutin as biomarkers in soils [64]. Estournel-Pelardy et al. used a two-step derivatization method to selectively analyze specific biomolecules including suberin-derived species present in peat [65].

Pyrolysis metastable atom bombardment time-of-flight mass spectrometry (Py-MAB-TOF-MS) is a fingerprinting method that has been used to analyze lipids in soils that originate from a variety of sources in an effort to expand the profile range of species and hence variability detected amongst different soils [66]. Pyrolysis-molecular beam mass spectrometry (py-MBMS) has also been used similarly as a fingerprint method to analyze lipid components in soils that could potentially be adapted specifically to suberin analysis as well [67, 68].

Liquid and solid-state NMR techniques have also been used to probe suberin architecture as well as compositional and structural information related to the specific constituents that comprise suberin biopolymers. Early solid-state NMR measurements first from potato skins, and later on cork suberin, revealed two distinct methylene CH_2_ environments within the aliphatic moieties at different chemical shifts with different dynamics properties [69–71]. It was proposed that the more motionally hindered methylene carbons are dense in –CH_2_–O– groups and might be physically closer to ester linkages [71]. As a clear demonstration of this observation that two distinct methylene CH_2_ domains exist, Yan and Stark used two-dimensional ^1^H–^13^C Wide-Line SEparation (WISE) NMR to study dynamics and domain architecture within wounded potato tissue [72]. The WISE experiment correlates the ^13^C chemical shift in the direct dimension with the ^1^H profile in the indirect dimension, thus providing insight into the rigidity as measured by the shape of the ^1^H profile. The majority of water-hydrated suberin remains rigid, but about 20% of its CH_2_ groups are quite dynamic with molecular motion in the 50 kHz frequency range [72].

Lopes et al. characterized extracts from cork suberin obtained from sequentially harsher alkaline methanolysis using GC/MS, ^1^H solution-state and ^13^C solid-state NMR techniques [73]. Together, results showed that suberin hydrocarbon chains that are most easily extracted (mild methanolysis conditions) are dominated by saturated 1-alkyanols, alkanoic acids, and *α*,*ω*-alkanedioic acids, whereas aliphatic components that require harsher methanolysis conditions are richer in mid-chain modified *ω*-hydroxyalkanoic acids. The findings also help explain the observation of two distinct methylene CH_2_ domains; it was proposed that a more rigid, partially ordered and repeating aliphatic acylglycerol region with various mid-chain modifications comprises a dominant central aliphatic structure, while hydrocarbon chains protrude into less ordered regions including the ferulate-rich polyaromatic region. Mild methanolysis conditions show that mostly *ω*-hydroxyacids and ferulates are released, but when harsher conditions are applied, mid-chain modified fatty acids are identified. Later, the stereochemistry of mid-chain modified hydroxyacids (9,10 epoxy and 9,10 diol groups) were identified using solution-state NMR [74].

Key solid-state NMR measurements have helped elucidate the spatial distributions of suberin subdomains. When suberized potato tissues are extensively solvent-extracted and enzymatically digested to remove unbound sugars and waxes, solid-state NMR data shows clear evidence of recalcitrant structural sugars, which are suggested to be bound to suberin [70, 75–77]. Arrieta-Baez and Stark show that these suberin-bound cell wall polysaccharides, which were consistent with cellulose-like and xylopyranose-like sugars, can be further removed under mild trifluoroacetic acid conditions [78]. Moreover, two separate WISE NMR spin-diffusion studies both suggest close spatial proximity of aliphatic carbons with both polysaccharide moieties and phenolic groups [72, 79]. These through-space findings were supported by high-resolution magic-angle spinning (HR-MAS) data of DMSO-swollen materials consisting of suberin and suberin-related species; 1D and 2D ^1^H and ^13^C HR-MAS data provide evidence of covalent linkages between polymers.

Like other biopolymers, improvements in analytical technologies for suberin are important for biomass optimization efforts both for its impacts on plant and ecosystem health but also in regard to its impacts on biomass designed for applications in renewable energy and chemicals.

### Suberin in biomass: a consideration in conversion to bio-products

The presence and structure of suberin in biomass clearly impacts plant growth, composition and survival and potentially has ecological ramifications on soil composition and health, including soil microbial composition. Additionally, suberin has implications on biomass conversion platforms related to its role in impacting the yield of desired products either due to the direct role of suberin abundance and structure on lignocellulosic feedstock conversion efficiency and/or in its indirect role impacting the productivity and composition of biomass used for biochemical or bioenergy production. As lignin composition in belowground biomass may have a relationship with total biomass yield of aboveground tissue that is used in conversion processes, and may also impact the ability of a feedstock to sequester C in soil [80]; suberin deserves dedicated focus for similar impacts on aboveground biomass production and C sequestration, particularly as it may otherwise be included as part of the lignin fraction during biomass characterization. Additionally, the presence of suberin in biomass such as waste food and agricultural residues may impact the yield and production of renewable chemicals and energy, but studies on this hypothesis are lacking. Lastly, suberin itself may be valorized as a resource for renewable, bio-derived energy and chemicals [1, 81–84].

**Fig. 1:**
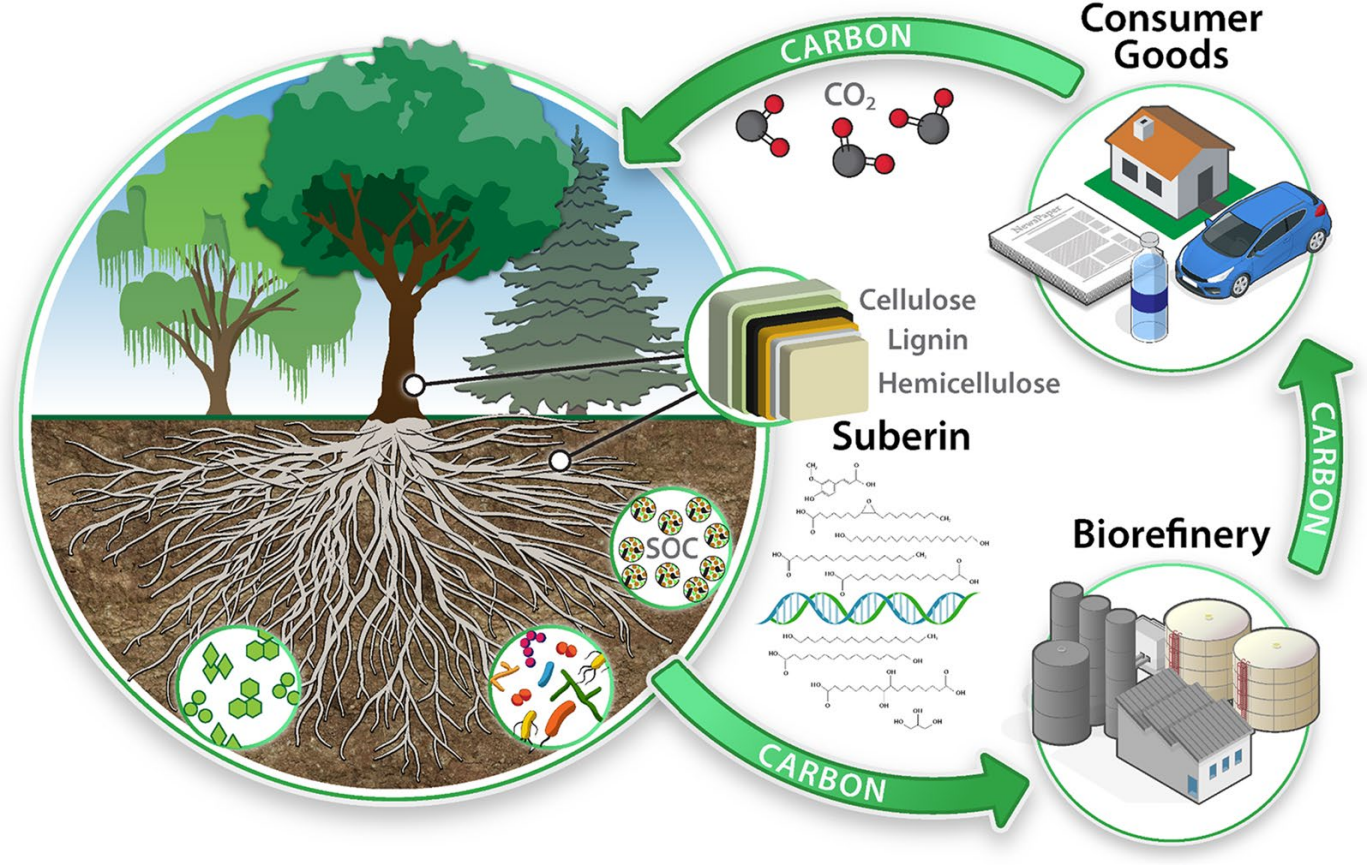
Graphical representation of the significance of suberin in plant performance (plant function context), belowground C inputs (ecosystem and biogeochemistry context) and valorization of C in biomass for bio-derived energy and materials (economic context). Greater throughput and accuracy in suberin analytical technologies and a greater understanding of molecular controls of suberin biosynthesis pathways are needed to enable informed and sustainable crop improvement strategies and realize a circular carbon-neutral bioeconomy

Due to its important role in the health and sustainability of plants, crop systems, and ecosystems as well as its own valorization potential and impact on conversion processes, it is imperative that suberin production in plants is considered in designing and cultivating crops intended for biochemical, bioproduct and bioenergy production (Fig. 1). Here we review the state of science and technology associated with suberin production and characterization in plants, its potential role in soil C inputs and impacts on biomass conversion processes. To cover the breadth of the subtopics within the complex subject of suberin chemistry and biology, we have attempted to highlight primary research works and comprehensive reviews (avoiding in- depth discussion of biosynthesis pathways of waxes, lignin and suberin, for which excellent literature have been published). We also aim to capture exemplary research approaches and statuses of insights. Furthermore, we broadly synthesize the current state of knowledge and provide perspectives on the importance of suberin and the merits of advanced biomass optimization efforts towards plant function, economic and environmental benefits.

## State of science and technology

### Genomics and genetic studies of suberin biosynthesis

Studies employing plant genetic variants or mutants and characterization of associated suberin pathway or phenotype modification(s) have been foundational for understanding and validating the physiological importance of suberin in plants [7]. Another complementary approach to identifying candidate genes underlying suberin biosynthesis and deposition has been via contrasting gene expression and metabolism in plants under ambient vs. treated/modified growth conditions to identify extended molecular networks of adaptation [85, 86]. Plant variants, mutants, enzyme biochemistry and environmentally controlled/perturbed studies have been integral to understanding of genes, genetic networks and biosynthetic pathways associated with suberin composition, biosynthesis, spatiotemporal regulation, deposition, and functional significance of suberin. There are several biosynthetic pathways involved in production of the monomers of suberin, a complex heteropolymer. These involve hydroxylation of fatty acids, oxidation to dicarboxylic acids, fatty acid elongation, reduction or extension of fatty acyl chains to primary fatty alcohols, glycerol acylations, incorporation of phenolics, amongst other processes used to produce suberin in tissues [87]. Table 3 summarizes select genes that have been identified in playing a role in suberin production and composition in plants.

**Table 3:** Summary of select genes shown to impact suberin production and structure in plants

Gene symbol	Plant type	Effect on suberin/plant phenotype	Reference
*AtMYB107, AtMYB9*	*Arabidopsis thaliana*	Regulates suberin synthesis in seed coats	[116]
*ANAC046*	*Arabidopsis thaliana*	Regulates suberin accumulation in roots	[175]
*AtMYB41*	*Arabidopsis thaliana*	Regulates for aliphatic suberin synthesis and impacts lamellae structure	[118]
*DAISY/KCS2, KCS20)*	*Arabidopsis thaliana*	Biosynthesis of cuticular wax and root suberin. Potentially redundant functions of genes	[97, 98]
*KCS1*	*Arabidopsis thaliana*	Synthesis of very-long-chain fatty acid (VLCFA) products in multiple wax biosynthetic pathways	[93]
*CYP86A1*	*Arabidopsis thaliana*	Aliphatic root suberin biosynthetic enzyme	[88]
*CYP86B1*	*Arabidopsis thaliana*	Suberin aliphatic monomer (very-long-chain saturated α,ω-bifunctional) biosynthetic enzyme	[90]
*FAR1, FAR4, FAR5*	*Arabidopsis thaliana*	Impacts root and seed coat suberin composition (C18:0-OH in *far5-1*, C20:0-OH in *far4-1*, and C22:0-OH in *far1-1*, mutants)	[99]
*GPAT5*	*Arabidopsis thaliana*	Impacts aliphatic suberin quantity in roots and very long chain dicarboxylic acid and ω-hydroxy fatty in seed coats	[120]
*ABCG2, ABCG6,* and *ABCG20*	*Arabidopsis thaliana*	Impacts effective suberin synthesis/production in roots, seed coats and pollen wall	[176]
*StNAC103*	Potato and *Arabidopsis*	Regulates suberin and wax deposition and formation of tuber apoplastic barriers	[177]
*QsMYB1*	*Quercus suber*	Regulates several biosynthesis and transport genes in suberin and lignin pathways	[178]
*MdMYB93*	Apple	When heterologously expressed in tobacco leaves, regulates accumulation of suberin as well as precursors of suberin and lignin	[117]
*CYP86A33*	Potato	Enzymatic functionalization of suberin aliphatic compounds at ω-terminal C end in periderm	[91]
*DSO/ABCG11*	*Arabidopsis thaliana*	Impacts suberin composition in roots and cutin biosynthesis aboveground	[179]
*AchnABF2, AchnMYB4,* *AchnMYB41, AchnMYB107*	*Actinidia chinensis* (kiwifruit)	Regulates suberin biosynthesis genes and suberin monomer accumulation	[180]
*SUBERMAN (SUB)*	*Arabidopsis thaliana*	Regulates suberin pathway genes and lamellae formation	[51]
*LTPG15*	*Arabidopsis thaliana*	Transport protein involved in very-long-chain fatty acids transport for suberin production	[181]
*ASFT (BAHD family)*	*Arabidopsis thaliana*	Feruloyl transferase impacts suberin-associated ferulate abundance	[100]
*RWP1 (HHT/BAHD family )*	*Arabidopsis*	Reduction of ω-hydroxyacid:hydroxycinnamoyltransferase level/activity reduced ferulate content of suberin. Impacts composition (ferulate) of suberin in root, stem, and seed	[101]
*FACT*	*Arabidopsis*	Impacts alkyl caffeate levels in suberized tissues	[4]
*FHT*	Potato	Impacts ferulate esters levels, altered developmental and water permeability properties	[103]
*PtFHT1*	*Populus*	Heterologous expression in *Arabidopsis* results in higher root ferulate levels but not p-coumarate	[104]
*THT* (*tyramine N-hydroxycinnamoyltransferase)*	Tobacco, Potato, Pepper	Enzymatic synthesis of feruloyltyramine	[105–108]
*ABCG1*	*Arabidopsis* and Potato	Impacts suberin barrier formation in roots and tuber periderm (potato) and accumulation of suberin precursors	[112, 113]
*At2g28670/ (esb1 mutant) *	*Arabidopsis thaliana*	Increased suberin levels in roots of loss-of-function mutant	[22]
*AtMYB92*	* Arabidopsis*	Regulates fatty acid and suberin biosynthetic genes and production of suberin monomers in tobacco leaf assays	[182]
*SGN3*	*Arabidopsis*	Receptor-like kinase with role in membrane microdomain formation in endodermis impacts Casparian strip formation and lacks parallel enhancement in suberin deposition as a compensation mechanism	[183]
*ShMYB78*	Sugarcane	Shown as activator of suberin production in heterologous assay	[119]

Using a transcriptomics analysis approach, root-expressing genes belonging to the cytochrome P450 fatty acid *ω*-hydroxylase CYP86 and CYP94 subfamily were proposed to be involved in catalyzing fatty acid *ω*-hydroxylation. Experimental validation studies showed that *CYP86A1/HORST* is expressed particularly within suberized tissues of roots and further alterations in suberin observed in compositional analysis of the mutant demonstrated the gene’s involvement in suberin biosynthesis [88]. An observed 60% reduction in total root suberin was attributed to a reduction in carbon chains C16 and C18 oxygenated fatty acids in suberin from the *CYP86A1/(horst)* gene mutant, providing evidence of genetic control on root suberin levels [88, 89]. *CYP86B1* gene is characterized as having a similar expression pattern in the root endodermis, and the corresponding *ralph* mutant shows a monomer specific alteration of very long chain *ω*-hydroxy acids, diacids, although total suberin content was not significantly affected [90]. Based on this compositional insight, it has been proposed that *CYP86B1/RALPH* encodes a very long chain fatty acid (VLCFA) *ω*-hydroxylase in plants [90]. In a separate study, the periderm from tubers of *Solanum tuberosum* down-regulated in *CYP86A33* gene expression was found to be more fragile compared to control plant, and the RNAi-downregulated lines were also reported to have a reduction in weight, by 50% [91]. Plants down-regulated in *CYP86A33* gene had alterations in suberin ultrastructure showing a significant reduction in the thickness of suberin, the secondary wall of the periderm, and a significant decrease in *ω*-functionalized monomers in aliphatic suberin which correlated with disappearance of the characteristic alternating dark and light lamellae [91].

 Fatty acid elongation involves β-ketoacyl-CoA synthases (KCSs) [92–96]. There are three C2 extending fatty acid elongation cycles needed for the C24 backbone (acting as the longest carbon backbone chain) of *Arabidopsis* suberin monomers. Seven *KCS* genes have been described as having a prolific and specific expression in various tissues/organs including roots [92]. In *Arabidopsis*, at least five *KCS* family genes have been associated with elongation of very long chain monomers in root suberin (C22) [7]. The mild to moderate phenotypic effects observed in mutants corresponding to *KCS2/DAISY* and *KCS20* genes allude to potential redundancy within the 21-membered KCS family of *Arabidopsis*, a likely involvement with other bioprocesses requiring very-long-chain fatty acids and in turn, an impact on the fatty acid pool for biosynthesis of the suberin monomer [97, 98].

Knowledge of these empirically validated suberin biosynthesis pathway genes has allowed for new validated connections/candidate genes identified via network analysis, such as the fatty acyl reductases (FAR); FAR1, FAR4, and FAR5, and feruloyl transferases (ALIPHATIC SUBERIN FERULOYL TRANSFERASE (ASFT) and RWP1/HHT (*ω*-hydroxyacid:hydroxycinnamoyltransferase) belonging to the BAHD family of acyltransferases) [7, 99–101]. *Arabidopsis* knockout lines of FATTY ALCOHOL:CAFFEOYL-CoA CAFFEOYL TRANSFERASE (FACT), an acyltransferase closely related to ASFT, was reported to be dramatically reduced in alkyl caffeate content while alkyl coumarate content was unaffected in suberized tissues [4]. A salt-stress response role for FACT was also proposed. Furthermore, this study by Kosma et al. [4] to biochemically characterize FACT as well as FAR1, FAR4 and FAR5 enzymes suggested that distinct acyltransferases may have distinct affinities for coumarate, caffeate or ferulate group of alkyl hydroxycinnamates. Also, enzymes such as those integrating the fatty acid elongation (FAE) complex and the FAR pathway have been shown to be important for suberin biosynthesis [86, 99, 100].

Presence of a high degree of hydroxycinnamic acids and their derivatives such as feruloyltyramine distinguishes polyaromatic components of suberin from lignin. A complex network of feruloyl transferase and conjugation enzymes catalyze phenylpropanoid pathway-derived ferulate (p-hydroxycinnamic acid) [102] and tyrosine-derived tyramine and their integration. Downregulation of a feruloyl transferase (*FHT*) gene via RNAi in potato periderm caused a reduction in ferulate esters, impacted developmental and functional water permeability properties, however, lamellar structure was apparently unaffected [103]. Ectopic expression of *Populus PtFHT1* gene in *Arabidopsis* resulted in higher root ferulate levels but not *p*-coumarate [104]. Distinct from previously mentioned significance of BAHD family of acyltransferases established via genetic studies, tyramine N-hydroxycinnamoyltransferases (THT; hydroxycinnamoyl‐CoA:tyramine N‐hydroxycinnamoyltransferase) have also been cloned and biochemically validated from multiple species such as tobacco [105], potato [106, 107] and *Capsicum annuum* (heterologous expression in rice) [108] to be important in synthesis of feruloyltyramine, a key component of suberin, and a concomitant differential regulation in response to wounding in potato [109]. Mutant studies in *Arabidopsis* have led to identification of an enhanced suberin mutant, *esb1*, opening up the potential and promise of the approach in discovering additional suberin pathway genes from among the endodermis specific genes [22, 110]. The corresponding *ESB1* gene locus, At2g28670, is expressed within the endodermis [22]. The *esb1* mutant roots had a two-fold increase in suberin aliphatic content and a disordered Casparian strip relative to control roots [19, 22]. The characterization of *esb1* has led to improvements in understanding the relationship between suberin content and root permeability, providing the first genetic evidence of suberin’s role in both ion translocation to shoots and water balance [7, 22]. Future analyses of modified transgenic lines expressing *ESB1* gene, driven by tissue specific or designer promoters and use of advanced analytical technologies can deepen the understanding of the role of suberization and formation of Casparian strip barriers in plant nutrient acquisition, growth and biomass quality and productivity, as well as advance knowledge on the genetic underpinnings.

Membrane proteins of the ABC family (ABCG2, ABC611, ABCG15, ABCG16, and ABCG32) are shown or suggested to aid in the transport of suberin monomers through the plasma membrane [86, 111]. Suberin composition is impacted in *Arabidopsis atabcg1* mutants (mutations in ABC transporter family gene, *ABCG1*) with a particular reduction in fatty alcohols and acids and long chain dicarboxylic acids [112]. A similar report of linking ABCG1 to suberin was obtained from potato tuber periderm studies [113]. Abscisic acid (ABA) can aid in suberin deposition in response to plant tissue wounding and abiotic stressors, or by application of the phytohormone [114]. Potato NAC protein StNAC103, a putative ortholog of *Arabidopsis* ANAC058, has been shown to impact suberin deposition, though the target/direct downstream genes in this process have yet to be uncovered [115].

A number of *MYB* family genes have been shown to act on or regulate suberin. Roles of AtMYB107 and AtMYB9 in suberin biosynthesis pathway are well documented [116], as well as role of MdMYB93 in suberization in apple fruit skins [117]. MYB41 has been shown to regulate suberin accumulation when overexpressed in leaves, and is upregulated in root endodermis under abiotic stressors [118]. Conservation of expression context of orthologous *MYB93* genes from rice, tomato, apple, potato and grape suggests potential cross-species conservation of functional roles in suberin synthesis [116]. A MYB family member from sugarcane, ShMYB78, was recently shown to be an activator of suberin production [119]. Tobacco leaf transient expression studies using ShMYB78 showed ectopic deposition of suberin and upregulation of suberin biosynthesis genes.

SUBERMAN (SUB) transcription factor has been shown to increase the root suberin lamellae formation. In *Arabidopsis*, SUB has a regulatory role by transactivating promoters of suberin biosynthesis genes [51]. Beyond the effect of SUB transcription factor in regulating suberin biosynthesis genes, it can also effect expression and localization of suberin transporters, and in turn impact root physiology, nutrient/water uptake capacity and structural stability [51].

Studying suberin and wax composition through four developmental stages of hybrid *Populus* stem periderm led to the discovery of several candidate suberin pathway genes [86]. Chemical components of poplar bark periderm, *viz.*, suberin, lignin, and other surface waxes were characterized at four developmental stages [86]. Microscopy of bark tissue layers, stages 1 through 4, was used to correlate structural/anatomical changes, i.e., increased number of suberized cell layers, with suberin chemistry and cork maturity. Chemical analyses showed an increase in suberin monomer load with bark age [86]. Some genes, including *CYP86A7*, were exclusively expressed in developed stage 3 of cork. Transcriptome analyses showed that this stage corresponds to highest number of genes responding to FAE, wax biosynthesis and lipid polyester biosynthesis [86]. The study suggested that poplar homologs of cutin pathway enzymes can potentially catalyze oxidation of suberin aliphatics within tree bark [86]. In addition to the poplar homolog to the known GPAT5 enzyme, homologs to *GPAT* *6*, *7*, and *8* genes, which encode cutin-specific acyltranferases in *Arabidopsis* were also upregulated in *Populus* bark transcriptomes, possibly playing functional roles within the *Populus* suberin biosynthesis pathways [86, 120]. Expression of putative *Populus* homologs of *Arabidopsis* SHINE1 (SHN1)/WAX INDUCER1 (WIN1) [121], regulator of cutin and other aliphatic waxes biosynthesis, in older bark development stage tissues, suggests a potential role in *Populus* periderm suberization [86].

Genomics and genetic studies have shown that modification of genes involved in suberin biosynthetic pathways can significantly alter suberin composition and structure and have implications on plant growth and stress adaptability. Further expansion of the fundamental suberin biosynthesis knowledge base and integration with validations in bioenergy crop species under applied economic and environmental contexts will be useful towards sustainable bioenergy crop improvements efforts.

### Understanding the relationships among plant suberin chemistry, soil C and the environment

Various studies support suberin’s potential implications on carbon (C) input/storage/persistence belowground and on soil organic matter and soil properties such as aggregation which may in turn relate to biomass decomposition and microbial interactions below ground [12, 16–18, 60, 64]. Increasing root biomass particularly with more fine roots and deeper roots is considered a complementary approach to increasing plant’s ability to capture, convert and allocate more C from the atmosphere to belowground [15, 122, 123]. Additionally, soil in heavily tilled farmlands are depleted in C reserves. Having the ability to return C inputs to soil has the benefits of improved soil health and plant productivity in addition to the C sequestration in soil for longer decadal time frames.

Fine root turnover as well as root exudates contribute to large inputs of organic C into soil, which supports soil health and microbial diversity, and in turn plant growth and biomass productivity, in a feedback loop [15, 124]. Roots can have variable ratios of relatively labile (sugars and carbohydrates) to relatively degradation resistant (complex polymers such as suberin and lignin) C forms and therefore, root chemistry and depth along with soil type and management are major factors in determining physical aggregate structure and microbial interactions, and ultimately the decades-long residence time of C or sequestration in soils. For example, fine roots can change their specific root area, length, diameter, density and chemistry, in order to improve resource uptake, and in response to external environmental changes such as elevated water availability and elevated CO_2_ [125–127]. A study in grasses showed that elevated CO_2_ levels and temperature can result in increases in suberin content by 28%, and by 36%, respectively [15]. These results can be attributed to the above- and below-ground morphological and physiological changes that have been reported from warming and elevated CO_2_ studies [15, 126]. Elevated CO_2_ coupled with environmental warming can result in greater specific root length and specific root area, and potentially increase the content of suberin per unit of mass [15, 126, 127].

While it is known that soil organic C has horizontal (along rooting path) and vertical (rooting and soil depth paths) gradients, an important aspect in understanding the decomposition of organic C in soil is to distinguish between above- and below-ground contributing sources. C source can be distinguished, relatively, in studies tracing cutin and suberin as suberin is primarily root-derived, while cutin is primarily leaf-derived. Based on a study that used field and lab incubation experiments to track contributions of plant biopolymers to SOM, it was reported that in the deciduous forest type studied, relative to leaf, root-contributed aliphatic compounds are a source of SOM with greater stability [17]. As summarized in the following additional examples, studies connecting suberin to SOM and C stabilization and storage belowground vary substantially in scale and resolution in the plant systems studied (forest type, crop system type or cultivar influences, etc.), analytical methods employed and whether cutin, lignin and suberin were differentiated from each other or not. In one study, carbon-14 (^14^C) suberin molecular markers were used, which correlated with root biomass [128]. A positive correlation was observed between SOC and ^14^C content with fine root necromass, which suggested their greater contribution to SOC, in part due to suberin, and also that root necromass acts as a major source of SOC at soil depths greater than 60 cm. The weaker correlation between suberin and root necromass in surface soil profiles (between 10 and 35 cm deep) may be attributed to a higher level of degradation of root biomass, and a lower suberin content [128]. In another report by Angst et al. [129] focusing on a similar concept, a two phase model was proposed based on decomposition of suberin and cutin, using mass loss and NMR measurements. Rapid mass loss of suberin and cutin monomers was found to occur at the beginning of the incubated experiment due to the ability of soil microorganisms to rapidly degrade suberin and cutin disassociated with lignin, but a steady maintenance was observed for the latter half of the decomposition study. It was hypothesized that the slower, steadier decomposition in the latter half of the study was due to the recalcitrance of the residual lignin coupled with suberin and/or cutin monomer. A large part of variation seen among lipids, however, was not associated with assessed factors [129]. The study conducted focused upon fresh root as well as leaves and needles from European Beech and Norway Spruce, respectively [129]. In agreement with the other study by Angst, belowground sources or roots were more recalcitrant than aboveground sources including leaves and needles, the latter can be linked to the higher availability of more easily degradable substances [128, 129]. To gain insights into the coupling effect of lignin with suberin and cutin, effects of specific cutin and suberin monomers, chain length and lipid type were tested [129]. An inverse relationship between lipid concentration and chain length (C atoms within each monomer) was observed [129]. In conjunction with this information, slower mass loss of roots when compared to leaf and needle material suggests that suberin monomers (containing fatty acids with chain length greater than C20) may  potentially decompose slower than cutin monomers (containing acids with chain length less than C18) [129]. These studies also suggest that α,ω-alkanedioic and mid-chain hydroxy acids can be used as root-specific markers and as shoot-specific markers, respectively [129, 130]. Even with the knowledge of these quantified factors, there remain other understudied influences such as relationships among variation in lipid concentration, organic mineral interactions, and their co-metabolism [128, 129] and their linkages to plant genetics is also understudied.

A study by Sumiyoshi et al. [80] aimed to address relationships between above- and below-ground biomass yield with variations in biomass composition and soil organic C pools. Their study, based on different types of perennial grasses, suggests root lignin content to be a primary driver in the rate of decomposition of plant tissues. Additionally, they found a correlation between aboveground and belowground biomass although this did not translate to higher soil organic C pools. Higher decomposition rates of plant tissues aligned with lower lignin composition in the biomass, but the authors did not separately account for suberin [80]. Similarly, studies are needed with a focus on suberin composition and structure in above- and below-ground tissues of bioenergy-relevant feedstocks and their resulting C inputs in the soil and impacts on C transformations and sequestration in soil, in order to be able to understand, quantify and model implications of suberin.

In a study of rice and the rape crops rotation, bulk and rhizosphere soil samples were analyzed for suberin diacids using GC–MS and compared to infer differences in C inputs across growth stages and cultivar types [131]. The study found that the monomer composition of suberin was altered across growth stages in a cultivar type. Suberin-derived monomer levels were higher in root rhizosphere relative to bulk soil, which also significantly correlated with soil organic C, SOC. The turnover and persistence of these suberin compounds in soil was, however, not followed in this short-term study [131].

While the fundamental genetics and genomics studies have yet to foray into implications in ecosystem settings and interconnections among the relevance of suberin in C contributions to soil, exciting recent discoveries in suberin biology such as discovery of a key suberization regulator [51] and evidence for reciprocal effects of root suberin (endodermal function) and associated microbiome [27], and the rapidly expanding genetic optimization approaches present exciting new avenues for addressing climate change challenges. Optimizing suberin and lignin content and composition in plant roots, increasing total root surface area, and creating deeper, more recalcitrant root systems could improve crop productivity and resilience while capturing and storing more C belowground.

### Conversion of suberin and suberin-rich biomass to bio-products

Biological and thermochemical conversion of lignocellulosic biomass focuses primarily on methods used to convert and valorize the biopolymer cell wall components lignin, cellulose and hemicellulose. Suberin occurs in specialized tissues and certain types and physiological fractions of biomass including roots and bark that may occur in abundance in forestry and agricultural waste streams. Relevancy of bark and the significant suberin component in biomass harvested from woody bioenergy feedstock crops have received limited attention relative to lignin and cellulose. The indirect impacts of suberin abundance and composition on stem biomass conversion are related to the effects of root and/or bark suberin variation on plant growth, physiology and chemistry including composition of lignin, cellulose and hemicellulose or on overall agronomic performance (sustainable growth, stress adaption, yield agricultural inputs, etc.) of the feedstocks [80]. The direct impacts of suberin present in biomass conversion processes are related to the contribution of suberin as a component that produces favorable or unfavorable bioproducts or impacts the yields of the products from certain processes. Additionally, suberin has lower oxygen content (< 15 wt%) and higher energy content relative to wood (24 vs. 21 MJ/kg) potentially making it an amenable feedstock for conversion processes [132]. Reviews covering specific routes and applications of bark and suberin conversion, particularly to renewable materials such as resins and composites can be found in [1, 84].

Thermochemical conversion methods such as pyrolysis are used to convert biomass to solid, liquid and gaseous products that could be used for chemical and energy production. Various types of catalysts and process conditions can be used to tune the distribution and properties of products derived from biomass where the biomass composition and pretreatment considerations are key factors in the conversion methodology. Thermochemical conversion of biomass high in suberin content relative to low-suberin biomass may result in various property and compositional differences related to water content, high-heating value and specific lipid-derived species present in bio-oils. For example, silver birch bark pyrolyzed in a series of thermal stages and subsequent fractionation generated a variety of suberin-derived products in the organic fractions but overall lower liquid yield (37.1 wt% vs. 60–65 wt%) and higher yields of certain oxygenated compounds such as fatty acids and aqueous fractions; which are substantially less favorable qualities in comparison to products generated from pyrolysis of lower suberin birch woody xylem [133]. Studies on the relative amount of bark in feedstocks have shown impacts on thermochemical conversion of blends that included pine residues with bark [134] and various properties of oils [135]. Ren et al. pyrolyzed mixtures of loblolly pine wood and bark where they demonstrated that pure bark and higher bark content mixtures produced less favorable oil characteristics including higher water (increasing in bark content mixtures of up to 20 wt% water in the bark oil) and oxygen content (oxygen content of wood oil being approximately 20 wt% and increasing with bark incorporation with pure bark oil being approximately 37 wt% oxygen) and phase separation (which was minimized at 50:50 mixture of wood:bark) [135]. However, they suggested it was important and possible to establish mixing ratios of wood:bark feeds to generate acceptable oils that valorize high-suberin waste or residual biomass feeds. Pyrolysis and gasification of pine park to produce syngas have also been investigated in co-processing mixtures with tire waste [136]. Synergistic effects were observed for the conversion of mixtures where co-pyrolysis enhanced energy efficiency and the addition of pine bark increased the quality of syngas generated from tire waste; for example, pine bark and waste tire mixtures resulted in higher C_m_H_n_ conversion than their respective individual fractions [136]. Also, balsam fir bark pyrolysis oil and extracts have been studied to elucidate antioxidant and enzymatic inhibition properties, particularly in relation to higher-value product streams [137].

Catalysts have been incorporated in other thermochemical routes such as hydrogenolysis and depolymerization processes that have been implemented on barks and suberin-rich materials. Garrett et al. performed catalytic hydrogenolysis on various types of biomass barks using two different catalysts to understand the chemistry associated with the production of lipid and aromatic species derived from the suberin and lignin in the barks [138]. Their study highlighted the differences in suberin depolymerization from different biomass sources, namely spruce, sycamore and cork. For example, cork produced the highest oil yield from hydrogenolysis using Rh/C (11.5 wt%) but the lowest oil yield using Pd/C (7.2 wt%) whereas the highest oil yield from Pd/C was generated from sycamore (13.3 wt%) [138]. Various types of fatty acids derived from suberin were produced in yields totaling 2–3 wt% in catalytic runs and aromatics were produced on the order of 1–4 wt% depending on catalysts, feeds and conditions [138]. In a follow-up study, McCallum et al. investigated the hydrogenolysis of cork in the presence of heterogeneous catalyst supported on various bases and in different solvents to optimize yield and environmental impacts of the proposed conversion routes [139]. The authors reported oil yields of up to 42.6 wt% where lipid yields were maximized in solvent of 2-methyltetrahydrofuran:water ratio of 6:4 and aromatic yields were maximized to 8.7 wt% in methanol [139]. *Quercus* bark (cork) has undergone reductive catalytic fractionation (RCF) for production of bio-oil and specific chemicals including 4-ethylguaiacol derived from lignin and suberin [83]. RCF has also been used to convert black locust bark and wood to oils and different valorization strategies were suggested based on the differences in product properties between the two feeds resulting from suberin conversion [140]. Bark oil was produced at a maximum of 35.1 wt% yield using Pd/C catalyst where phenolics were produced at approximately 3 wt% yield and aliphatic monomers were produced at approximately 9 wt% yield of the bark depending on the catalyst and conditions used [140].

Other non-biological conversion strategies such as acid hydrolysis and liquefaction have been used to convert barks to chemical intermediates and products. Two stage acid hydrolysis of birch wood and bark was investigated by Kim et al. [141] to find optimal conversion conditions for the production of fermentable sugars. Acid catalyzed liquefaction of eucalyptus bark to recover cellulosic and sugar-derived products was investigated by Mateus et al. [142]. However, the impact of suberin directly on these processes was not fully considered.

Direct thermochemical depolymerization of suberin has been used to produce biofuels particularly in order to take advantage of its high energy content. Kumaniaev et al. isolated and depolymerized suberin from birch bark in an optimized system and subsequently upgraded the oligomeric products by hydrotreatment to produce diesel and aviation fuel ranges [143]. Oil yield was 40 wt% of the original bark mass with average higher heating value of 46.5 MJ/kg and based on 2-D GC analysis the oil products consisted of approximately 24 wt% n-alkanes, 23% branched alkanes and 25% alkenes/cycloalkenes where benzenes and aromatics constituted the remaining fractions [143]. Many thermochemical conversion methodologies of bark and high-suberin materials have mostly focused on the impacts of inorganics present in bark, [144] which does complicate a fundamental understanding of the contribution of suberin in the bark conversion, but further focus on the suberin impacts needs to be expanded. Additionally, studies outlining intentional removal of suberin prior to biomass conversion (aside from debarking) and impacts on biomass residue and resulting conversion potential are lacking.

Biological conversion methods used to convert biomass include enzymatic saccharification and hydrolysis, fermentation and anaerobic digestion (AD). Like thermochemical processes, biomass composition and pretreatment are important variables that can impact the yield and type of products generated. Suberin and/or bark presence in biological conversion methods targeted for sugar-derived chemicals by enzymatic hydrolysis has generally been shown to negatively impact the yield of desired products. For example, black locust bark suberin with known biocidal activity [145] must be considered in microbial fermentation and enzymatic conversion of sugars and pretreatment strategies of biomass containing suberin [146]. It is also relevant to note that suberin has been shown to have significant impacts on the digestibility of sugarcane cultivated for animal forage [147, 148]. Enzymatic conversion of elephant grass bark was not as readily degraded relative to the pith possibly due to the presence of biopolymers such as cutin as studied by Perez-Boada [149]. Anaerobic digestion (AD) of potato peel waste and its fermentation residue was performed by Liang et al. concomitantly with various characterization methods to better understand the relationship between the presence of biopolymer components such as lipids derived from suberin in the feedstocks and resulting products after conversion [150]. The authors hypothesized that their feedstocks produced up to approximately 65% CH_4_ yield, higher than that produced from wood, in part due to the high lipid contents of the potato peel and corresponding fermentation residues (being 2–8 wt%). Utilization of *Pinus patula* bark in enzymatic saccharification and fermentation processes with implications on a biorefinery concept have been demonstrated, however, suberin was not specifically considered in the study [151].

Combinations of thermochemical and biological conversion platforms can improve economics and utilization of waste materials in biorefinery concepts. Like the individual approaches, combined conversion platforms may still be impacted and be necessarily adaptable to differences and changes in feedstock properties and composition. However, most studies have focused on feedstock quality attributes such as lignin content, ash content, cellulose crystallinity, surface area, etc. without considering suberin which would otherwise be related to bark content, energy content and other attributes known to impact pretreatment and conversion economics [152]. Rasi et al. demonstrated a cascade process of hot water extraction, AD and pyrolysis that could be used to valorize pine and spruce barks, but specific impacts of suberin on the processes were not covered [153]. Short rotation woody crop (SRWC) air classification was used to separate bark to improve combined bio-thermal conversion methodologies for conversion of “clean” woody material [154]. Their study showed that the whole biomass and “clean” wood (air classified to remove leaves, some bark, etc.) produced higher yields of pyrolysis oils with improved properties such as lower oxygen content than the “unclean” fraction consisting of bark and leaves, which produced higher amounts of char and gases [154], but suberin contribution to the processes or chemistry was not considered. A significant amount of work is still needed to better understand and improve conversion paradigms incorporating suberin chemistry from bioenergy-relevant feedstocks and the economic impacts on different processes. Additionally, demonstration of the effects of genetically modified stem suberin on these conversion processes is even less explored.

## Conclusions and future perspectives

### Knowledge gaps in understanding and optimizing suberin for sustainable bioenergy crop production

The significance of suberin in plant performance is unequivocal, and there are several lines of evidence supporting significance of suberin in potential economic (biomass conversion to energy and materials) and ecological (C inputs into soil and biogeochemical cycling) contexts. Substantial progress has been made in gaining genomics insights and developing analytical methods to understand suberin biosynthesis in plants and deposition in plants and soil. However, there is a need to increase the pace and expand the breadth and depth of these studies, particularly for bioenergy-relevant crops.

First, our current understanding of suberin genomics is derived primarily from plant growth and adaptation studies using *Arabidopsis* and food crops (Table 3) and is centered on linking gene function to suberin structure and function in plants. Accelerating suberin genomics/genetics studies in dedicated bioenergy crops that link suberin biology to agronomic, ecological and economic impacts will be needed to expand our understanding and practically consider suberin in sustainable bioenergy crop improvements efforts. Multi-omics strategies, as reviewed in [155], have been employed to understand structure–function relationships and how the cuticle layer and suberin lamellae are formed in many types of plants and tissues. However, such -omics strategies are yet to be employed towards understanding control-knobs of suberin chemistry in biorefinery-relevant lignocellulosic feedstocks (switchgrass, pine, poplar, etc.) along with co-considerations of sustainability metrics.

Co-considerations of above- and below-ground plant chemistry and productivity will be necessary, especially in the context of suberin. Studies show that there is a strong genetic component and cultivar specificity to suberin quality and quantity, while also showing that as part of plant’s adaptive mechanisms, suberin biosynthesis can be influenced by external abiotic and biotic factors. Applications of genomics/genetics and analytical approaches to characterizing stems and roots of large replicated populations under field conditions are needed to understand and quantify the interactive effects of the genetic and environmental components and improve crop performance for future climate scenarios. Advancements in systems and synthetic biology approaches can be leveraged to design plants with precise and differential gene expression in above and belowground tissues to generate plants that are co-optimized for enabling a carbon–neutral bioeconomy [156]. Extending plant-level suberin studies to crop plantation and stand levels for aboveground harvest and conversion metrics, and for belowground C budgeting and soil health metrics will be critical to addressing the large knowledge gaps between “the potential” and “the practical.”

Correlation of suberin chemistry to soil health, microbial activity, and persistence and sequestration of soil C needs to be evaluated using improved analytical technologies in order to quantify the content, structure, composition and significance of suberin [64, 129] and establish its genetic underpinnings. Future studies will, therefore, need to consider substantially longer timeframes in keeping with decadal timeframes for C sequestration processes.

### Technological advances needed for the analysis of suberin

While many wet chemistry, microscopy and spectroscopic techniques are used to isolate and/or analyze suberin in biomass successfully at various scales and with varying degrees of changes induced on the native structure and composition, there is not consistent or standardized and validated methodology that is universally used to characterize and define suberin content, structure and composition in biomass. Suberin architecture, spatiotemporal dynamics and macromolecular structure in cell walls are particularly primed for new advancements in knowledge. For example, in recent years multiple groundbreaking studies have applied multi-dimensional and other advanced solid-state NMR methodologies to ^13^C-enriched plant and fungal cell walls, gaining key information on their detailed molecular structure and high-level architecture [157, 158]. While as previously discussed ssNMR methods have proven invaluable, to the best of our knowledge advanced multi-dimensional ssNMR techniques, which could benefit from significant ^13^C isotopic enrichment, have not been applied to characterize suberized tissues. ^13^C enrichment of suberin should be possible, albeit expensive, by growing select plants in a ^13^CO_2_ atmosphere using a controlled growth chamber. Solution-state NMR methods should also be established for spectroscopic phenotyping. A few examples of HSQC NMR fingerprinting applied to suberin have been used by various groups, but established analytical protocols, comparable to those developed by the Ralph lab for lignin analysis are lacking [46, 59, 78, 159, 160].

The majority of suberin analysis methods require a number of steps to prepare samples and while analytical techniques can provide detailed speciation of suberin moieties, high-throughput suberin analyses for large sample populations are lacking. One possible solution would be to adapt high-throughput pipelines used to analyze sugars and lignin in biomass to analyze suberin content and/or composition [161]. It may also be possible to make straightforward and streamlined methods for simultaneous analysis of the components in suberin such as that outlined in Marques et al. [53] have higher-throughput with the use of rapid heating low thermal mass modular accelerated column heater (LTM-MACH) GC modules and/or incorporate robotics or automated sample handling. Quantitation of suberin-derived analytes in GC analysis may also be improved when standards aren’t available with the use of Polyarc reactors coupled to flame ionization detection (FID). Additionally, suberin analysis methods could aim to reduce the number of steps involved in the processes as outlined in Delude et al. [162]. Researchers could also benefit from development of rapid in-field analyses using hand-held spectrometers such as near infrared (NIR) or Raman, which have been used in lab or bench scale systems to analyze lignin content in roots [163] and in other biomass tissues [164].

Additionally, there are not specific, validated methodologies used for the characterization of suberin in soils, particularly to analyze isolated species that can differentiate biomass origins and are separate from microbial contributions, particularly lipid moieties [12, 13]. Suberin as a biomarker in soil has shown potential to be species specific [165] which may require that analytics be capable of resolving particular types of suberin from particular types of sources to properly inform relevant impacts of suberin from different biomass types on various sustainability metrics associated with feedstock production. An understanding of suberin chemistry in plant and soil health and those potential returns on biomass productivity and relationships with genomics and economics of conversion, as well as C utilization, is also needed for population-scale studies for feedstocks destined for biorefinery applications. The analysis of suberin-derived species at particular points in biorefinery processes will also be essential for understanding suberin impacts on lignocellulosic conversion and for identification of value-added components.

### Suberin for optimized conversion platforms and value-added bio-products

Optimizing suberin in biomass for conversion platforms can be approached by designing biomass with suberin that improves biomass yields, conversion potential and/or consists of suberin in plant tissues with favorable characteristics for direct valorization. Additionally, it will be important to establish relationships between biomass conversion and suberin abundance in bark, roots and other bioenergy feedstock tissues, with or without the presence of suberin in biomass being converted, thereby measuring the impacts of suberin on lignocellulosic conversion methodology. Further, conversion methods themselves can be optimized for biomass to include or account for suberin conversion.

Direct conversion of high-suberin biomass has been demonstrated using a number of different approaches as outlined here and in other reviews that have provided a brief history and review of some suberin utilization and conversion strategies for production of a variety of materials with various applications [1, 84]. High suberin biomass and isolated suberin conversion could potentially increase the utilization of biomass waste, particularly in biorefinery contexts. Incorporating suberin chemistry into genomics, biomass production and conversion platforms is desirable. Life cycle analyses (LCA) and techno-economic analyses (TEA) could be conducted to evaluate impacts of various suberin chemistry and associated plant performance scenarios on yield, titre, conversion approaches and other economic outcomes [143]. LCA and TEA using various scenarios of suberin incorporation can be useful in assessing outcomes on C budgeting (C capture vs release accounting) and the extended ecological and environmental impacts. The expansion of high-suberin biomass conversion and novel routes used to convert and valorize suberin itself could also ensure more efficient biomass resource utilization.

## Summary

Taken together, suberin exists at a high impact vantage point, and deeper and broader studies tracking suberin chemistry, underlying genes and associated economic and environmental impacts are urgently needed to undertake informed co-optimization of both above- and below-ground plant tissues and to enable the vision of a circular, carbon-neutral and sustainable bioeconomy. Harnessing plants and their chemistry for environmental and economic co-benefits will require us to address key gaps in our fundamental knowledge base, integrate above- and below-ground aspects and better model impacts across scales. Cross-disciplinary perspectives and expertise will be needed to cover plant biology, systems and synthetic biology, analytical chemistry, processing, agronomy, forestry, ecology, data analytics and modeling aspects for assessing and optimizing plant performance and productivity, and evaluating impacts on ecosystem and biorefinery performance. For population-scale studies and higher resolution characterization, there is a need for consistent, standardized and high-throughput analytical characterization with links to genome science and technology to enable predictive systems biology models. Last, but not the least, integration of suberin chemistry with multiple lines of evidence from genomics, phenotyping, biogeochemistry and conversion assessments into TEA and LCA models will be needed in holistically considered biorefinery operations and management of dedicated bioenergy crop plantations in order to enable a sustainable bioeconomy.

## Data Availability

Data sharing is not applicable to this article as no datasets were generated or analyzed during the current study.
